# Clinical Characteristics and Risk Factors Associated With Acute Kidney Injury Inpatient With Exertional Heatstroke: An Over 10-Year Intensive Care Survey

**DOI:** 10.3389/fmed.2021.678434

**Published:** 2021-05-19

**Authors:** Ming Wu, Conglin Wang, Zheying Liu, Li Zhong, Baojun Yu, Biao Cheng, Zhifeng Liu

**Affiliations:** ^1^The First School of Clinical Medicine, Southern Medical University, Guangzhou, China; ^2^Department of Critical Care Medicine, General Hospital of Southern Theatre Command of People's Liberation Army, Guangzhou, China; ^3^Department of Critical Care Medicine and Infection Prevention and Control, The Second People's Hospital of Shenzhen, First Affiliated Hospital of Shenzhen University, Health Science Center, Shenzhen, China; ^4^Department of Critical Care Medicine, The First Affiliated Hospital, Guizhou University of Chinese Medicine, Guiyang, China; ^5^Department of Critical Care Medicine, Bao'an People's Hospital, Shenzhen, China; ^6^Department of Plastic Surgery, General Hospital of Southern Theatre Command of People's Liberation Army, Guangzhou, China; ^7^Key Laboratory of Hot Zone Trauma Care and Tissue Repair of People's Liberation Army, General Hospital of Southern Theatre Command of People's Liberation Army, Guangzhou, China

**Keywords:** heatstroke, acute kidney injury, mortality, risk factors, SOFA

## Abstract

**Background:** Exertional heat stroke (EHS) is a life-threatening injury that can lead to acute kidney injury (AKI). The clinical characteristics of and risk factors for EHS complicated with AKI have been poorly documented.

**Methods:** A retrospective study with EHS admitted to the intensive care unit (ICU) from January 2008 to June 2019 was performed. Data including baseline clinical information at admission, main organ dysfunction, 90-day mortality and total cost of hospitalization were collected.

**Results:** A total of 187 patients were finally included, of which 82 (43.9%) had AKI. AKI patients had more severe organ injury and higher total hospitalization costs than non-AKI patients. Multivariate logistic analysis showed that lymphocyte, neutrophil, D-dimer and myoglobin (MB) ≥ 1,000 ng/ml were independent risk factors for AKI caused by EHS. In addition, SOFA score [hazard ratio (HR) 4.1, 95% confidence interval (95% CI) 1.6–10.8, *P* = 0.004] and GCS score (HR 3.2, 95% CI 1.2–8.4 *P* = 0.017) were the risk factor for 90-day mortality in patients with EHS complicated with AKI, with an area under the curve (AUC) of 0.920 (95% CI 0.842–0.998, *P* < 0.001) and 0.851 (95% CI 0.739–0.962, *P* < 0.001), respectively. Survival analysis showed that the 90-day mortality in AKI patients was significantly high (*P* < 0.0001) and the mortality rate of patients with AKI stage 2 was the highest than other stages.

**Conclusions:** EHS complicated with AKI is associated with higher hospitalization costs and poorly clinical outcomes. MB ≥1,000 ng/ml, Inflammation, coagulation were associated with the occurrence and development of AKI. Early treatment strategies based reducing the SOFA and GCS score may be pivotal for improving the prognosis of EHS.

## Introduction

Heat stroke is the most serious form of heat injury and is considered a life-threatening medical emergency requiring neurocritical care. In addition to primary brain injury, secondary multiple organ dysfunction syndrome (MODS), including acute kidney injury (AKI), is a major cause of death and disability in heat stroke patients (1, 2). According to the etiology, heatstroke can be classified as classic heat stroke (CHS) and exertional heatstroke (EHS) (1). Studies have shown that 25–35% of EHS and 5% of CHS patients develop acute oliguric kidney failure (3). AKI caused by heatstroke may be related to many factors, such as direct heat stress, prerenal injury caused by hypovolemia, renal insufficiency, rhabdomyolysis (RM), disseminated intravascular coagulation (DIC), and inflammatory reactions (3). There have been many studies on the clinical characteristics of and risk factors for kidney injury in sepsis patients, as kidney injury plays an important role in the treatment of sepsis (4). However, there have been relatively few large-sample clinical studies on EHS. To provide a reference for the timely and effective treatment of heatstroke complicated with AKI, a retrospective cohort study was designed in a tertiary-care teaching hospital in southern China over a 10-year period. The study mainly analyzed the clinical characteristics of, risk factors for and 90-day mortality associated with heatstroke complicated with AKI.

## Methods

### Study Design and Participants

This single-center retrospective cohort study was performed in the intensive care unit (ICU) of the General Hospital of Southern Theatre Command of PLA from January 2008 to June 2019. The inclusion criterion was as follows: patients with “heatstroke” caused by strenuous exercise performed in a high-temperature and high-humidity environment. The diagnostic criteria of heatstroke were as follows (1): exposure to a high temperature, high humidity or a history of strenuous exercise; a clinical syndrome causing an excessively high body temperature (central temperature higher than 40°C); nervous system dysfunction (including delirium, cognitive impairment, coma, etc.); or systemic organ dysfunction. The exclusion criteria were as follows: (1) death or discharged within 24 h after admission, (2) incomplete data regarding key indicators, (3) incomplete outcome evaluation data obtained *via* telephone follow-up, and (4) a previous history of organ dysfunction, such as chronic kidney disease.

The cause of heatstroke is strenuous outdoor training in southern China and all patients are treated with whole-body cooling as soon as possible and continue to cool down to 39°C (preferably 38.5–38.0°C) after admission. All patients received basic life support treatment according to their condition and were provided comprehensive treatment, including brain protection, anti-inflammation, administer fluids and organ function support, such as mechanical ventilation. Moreover, appropriate volume management was performed for patients with AKI, and renal replacement therapy such as continuous renal replacement therapy (CRRT) was initiated if necessary.

### Research Procedures

The basic characteristics of the patients were recorded, including ages, the Acute Physiology and Chronic Health Evaluation II (APACHE II) score, Sequential Organ Failure Assessment (SOFA) score, Glasgow Coma Scale (GCS) score, and inflammatory and organ function indicators at admission. The indicators included blood count (white blood cell, neutrophil, lymphocyte, monocytes, platelets, mean platelet volume, platelet distribution width), procalcitonin (PCT), C-reactive protein (CRP), liver function markers [total bilirubin, glutamate pyruvic transaminase (ALT), glutamic oxaloacetic transaminase (AST)], kidney function markers [blood urea nitrogen, serum creatinine (Scr), creatine kinase (CK)], cardiac markers [MB isoenzyme of creatine kinase (CK-MB), myoglobin (MB), cardiac troponin I (cTNI)], clotting factors [prothrombin time (PT), international normalized ratio (INR), activated partial thromboplastin time (APTT), thrombin time (TT), fibrinogen (FIB), D-dimer] and blood transfusion during treatment. All the patients were assigned to the AKI group or the non-AKI group according to the presence of AKI. The main results, including 90-day mortality, ICU time and the total cost during hospitalization, were analyzed, and survival curve analysis was performed.

### Definitions

1. AKI (5): KDIGO standard: Scr increase to ≥26.5 μmol/L (≥0.3 mg/dL) within 48 h, Scr increase to ≥ 1.5 times the baseline within 7 days, or urine output <0.5 ml/(kg∙h) for 6 h. The severity of AKI was divided into three different stages according to the following criteria. Stage 1 includes Scr increase 1.5–1.9 times baseline or ≥0.3 mg/dL (≥26.5 μmol/L) or urine output <0.5 ml/(kg∙h) for 6–12 h. Stage 2 includes Scr increase 2.0–2.9 times baseline or urine output <0.5 ml/(kg∙h) for ≥12 h. Stage 3 includes Scr increase 3.0 times baseline or Scr ≥ 4.0 mg/dL (≥353.6 μmol/L) or initiation of renal replacement therapy or in patients <18 years a decrease in estimated glomerular filtration rate (eGFR) to <35 ml/(min∙1.73 m^2^) or urine output <0.3 ml/(kg∙h) for ≥ 12 h or anuria for ≥12 h.
2. DIC (6): International Society for Thrombosis and Haemostasis (ISTH) standard: An ISTH score ≥ 5 points.
3. Acute liver injury (7): Serum total bilirubin ≥ 3.0 mg/dL, ALT > 41 U/L, or AST > 41 U/L.
4. RM (8): general fatigue, muscle soreness and soy sauce-like urine; elevated laboratory CK, and elevated non-cardiogenic MB. This study adopted the current consensus opinion that CK > 1,000 U/L or increased more than 5 times the normal level was considered elevated CK, while an increase in CK due to cardiogenic shock (CK-MB/CK <5%) was excluded.
5. Lymphocytopenia (9): absolute lymphocytes <0.8 ×10^9^/L.

### Statistical Analysis

The continuous variables conforming to a normal distribution are expressed as $\bar{x}\pm s$. Continuous variables that did not conform to a normal distribution, count data and ordinal data are expressed as median and interquartile ranges (IQRs). Count data were compared using multiple independent samples non-parametric Kruskal-Wallis H tests, and measurement data for intergroup comparisons were analyzed using non-parametric Mann-Whitney *U*-tests. Means of normally distributed measurement data were compared among multiple groups with single factor variance least significant difference (LSD) tests, and non-normally distributed data were compared with Kruskal-Wallis H tests. The patient-related endpoint was mortality 90 days after onset. The survival curve was drawn by R language. After proper adjustment for MB or CK, the difference in survival was evaluated with the logrank test. Significant indicators were analyzed using single factor analysis. Indicators with a *P-*value <0.1 were included in the multivariate logistic regression (LR) model, and forward stepwise regression was used to gradually eliminate each variable. The impact of each indicator on prognosis was analyzed, and prognostic risk factors were screened. The receiver operating characteristic (ROC) curve of each index was constructed by a non-parametric method, and the area under the curve (AUC) was calculated. The best diagnostic critical point was determined, and the sensitivity (SEN) and specificity (SPE) of each index in predicting mortality were calculated. Statistical analyses were performed using SPSS Windows version 23.0 (SPSS Inc., Chicago, IL), Empower (R) (http://www.empowerstats.com, X&Y solutions, Inc., Boston, MA) and R (http://www.R-project.org) software. *P*-values (two-tailed) <0.05 were considered statistically significant. The study was approved by the Research Ethics Committee of the General Hospital of Southern Theatre Command of PLA (HE-2020-09). In view of the retrospective study design and depersonalization of data, the Ethics Committee agreed to waive the requirement for patient written informed consent but required that the patients be informed of the study details during a telephone follow-up.

## Results

### Clinical Characteristics of Patients With EHS

**Figure 1:**
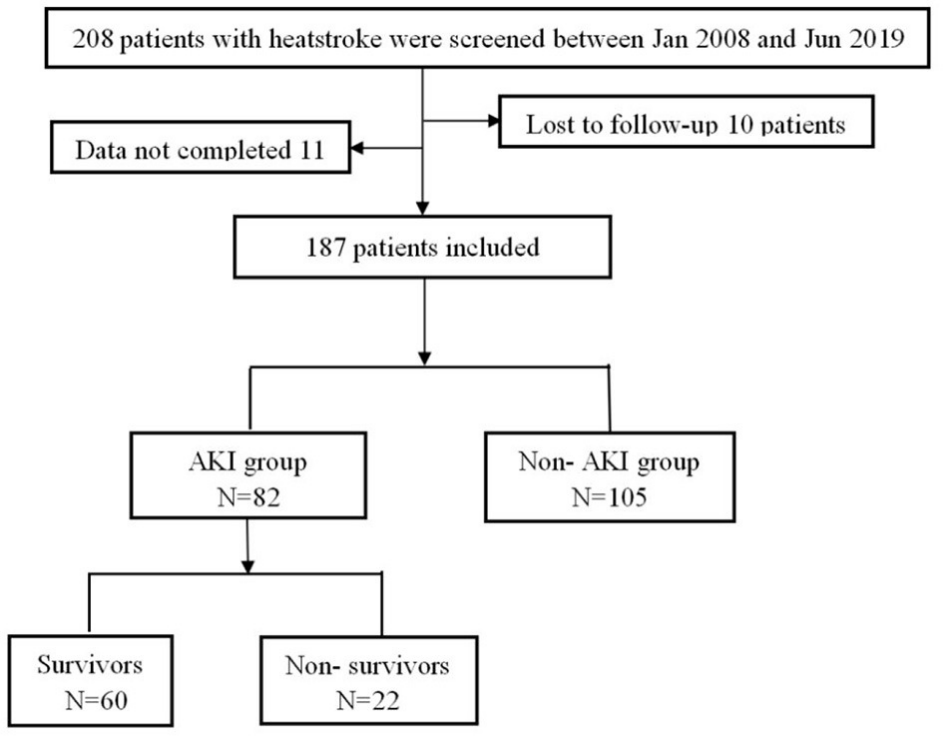
Flow chart of all excluded and included patients.

A total of 208 patients fulfilled the inclusion criteria, of whom 21 patients were excluded because they were lost to follow-up or were missing clinical data. Finally, 187 patients were included and all of them were males. Of these, 105 patients (56.1%) did not have AKI, and 82 patients (43.9%) had AKI (Figure 1). Compared with the patients without AKI, the patients with AKI had higher APACHE II (13.0 vs. 9.0, *P* < 0.001) and SOFA scores (5.0 vs. 2.0, *P* < 0.001); lower GCS scores (10.0 vs. 12.0, *P* = 0.008); and higher rates of MB ≥ 1,000 ng/ml (55.1 vs. 15.6%, *P* < 0.001), DIC (51.6 vs. 21.2%, *P* < 0.001) and acute liver injury (80.0 vs. 66.7%, *P* = 0.047). There were no significant differences in the incidence of lymphocytopenia (43.2 vs. 35.2%, *P* = 0.268) or RM (CK ≥ 1,000 U/L, 52.6 vs. 43.9%, *P* = 0.252). In addition, 90-day mortality in the AKI group was significantly increased (26.8 vs. 1.0%, *P* < 0.001), and the total cost of hospitalization was significantly higher [87,689.1 vs. 28,925.8 (RMB), *P* < 0.001] (Table 1).

**Table 1:** Comparisons of clinical characteristics between AKI and non-AKI patients with EHS.

	**Non- AKI (*n* = 105)**	**AKI (*n* = 82)**	***P*-value**
APACHE II score, median (IQR)	9.0 (7.0–14.0)	13.0 (9.0–21.0)	<0.001
SOFA score, median (IQR)	2.0 (2.0–4.0)	5.0 (3.0–9.0)	<0.001
GCS score, median (IQR)	12.0 (9.0–14.0)	10.0 (6.0–13.2)	0.008
Age (years), median (IQR)	20.0 (19.0–26.0)	23.0 (19.0–28.0)	0.063
WBC (1 ×10^9^/L), median (IQR)	10.3 (8.5–13.7)	11.9 (9.4–15.7)	0.010
Neutrophil (1 ×10^9^/L), median (IQR)	8.8 (6.3–11.8)	9.5 (6.7–13.2)	0.112
Lymphocyte (1 ×10^9^/L), median (IQR)	1.2 (0.7–1.8)	0.9 (0.5–2.4)	0.071
Monocytes (1 ×10^9^/L), median (IQR)	0.7 (0.4–0.9)	0.7 (0.4–1.0)	0.317
Platelets (1 ×10^9^/L), median (IQR)	174.0 (113.0–226.0)	113.0 (64.8–207.2)	0.001
Mean platelet volume (%), median (IQR)	10.7 (10.1–11.4)	10.7 (10.2–11.5)	0.861
Platelet distribution width (%), median (IQR)	12.5 (11.1–13.6)	12.4 (11.6–14.1)	0.254
TBIL (μmol/L), median (IQR)	15.4 (9.9–24.8)	16.5 (10.3–40.3)	0.238
ALT (U/L), median (IQR)	31.0 (17.0–97.0)	60.0 (25.0–847.0)	0.005
AST (U/L), median (IQR)	54.0 (29.0–133.0)	96.0 (44.0–422.5)	0.025
BUN (mmol/L), median (IQR)	4.9 (4.0–6.0)	6.8 (5.8–10.0)	<0.001
CR (μmol/L), median (IQR)	95.0 (78.0–119.0)	170.0 (149.0–228.0)	<0.001
Cystatin C(mg/L), median (IQR)	0.9 (0.8–1.0)	1.2 (1.0–1.8)	<0.001
CK (U/L), median (IQR)	793.5 (248.5–2,148.2)	1,110.0 (482.0–3,675.2)	0.034
CK-MB (ng/ml), median (IQR)	32.5 (22.0–59.8)	46.0 (29.2–101.8)	0.006
MB (ng/ml), median (IQR)	193.6 (63.2–583.5)	1,000.0 (469.9–1,000.0)	<0.001
cTNI (pg/ml), median (IQR)	55.0 (10.0–158.2)	300.2 (100.0–1,016.8)	0.015
PT (s), median (IQR)	15.4 (14.0–17.4)	17.7 (14.6–27.2)	<0.001
INR, median (IQR)	1.2 (1.1–1.4)	1.5 (1.2–2.5)	<0.001
APTT (s), median (IQR)	38.4 (34.5–43.6)	41.1 (32.8–78.6)	0.175
TT (s), median (IQR)	17.3 (16.5–18.4)	18.8 (16.8–29.2)	0.002
FIB (g/L), median (IQR)	2.6 (2.2–2.9)	2.3 (1.8–2.8)	0.015
D-D (mg/L), median (IQR)	0.8 (0.4–3.5)	5.3 (1.4–13.1)	<0.001
CRP (mg/dL), median (IQR)	3.3 (1.8–5.8)	3.4 (0.9–8.7)	0.543
PCT (ng/ml), median (IQR)	1.4 (0.7–3.6)	2.7 (1.2–5.3)	0.007
CK ≥ 1,000 U/L, *N* (%)	43 /98 (43.9%)	40/76 (52.6%)	0.252
MB ≥ 1,000 ng/ml, *N* (%)	14/90 (15.6%)	38/69 (55.1%)	<0.001
Transfusion *N* (%)	16/105 (15.2%)	35/75 (46.7%)	<0.001
Lymphocytopenia <0.8 ×10^9^/L, *N* (%)	37/105 (35.2%)	35/81 (43.2%)	0.268
DIC, *N* (%)	17/80 (21.2%)	33/64 (51.6%)	<0.001
Acute liver injury *N* (%)	66/99 (66.7%)	64/81(80.0%)	0.047
90-day mortality *N* (%)	1/105(1.0%)	22/82(26.8%)	<0.001
ICU time (d), median (IQR)	4.0 (3.0–7.0)	7.0 (4.0–14.0)	<0.001
Survival time (d), median (IQR)	90.0 (90.0–90.0)	90.0 (34.0–90.0)	<0.001
Hospitalization costs (RMB), median (IQR)	28,925.8 (18,132.1–49,212.0)	87,689.1 (40,293.0–188,228.3)	<0.001

### Risk Factors for EHS Complicated With AKI

The multivariate LR analysis showed that lymphocyte count (OR 2.2, 95% CI 1.2–3.4, *P* = 0.005), neutrophil count (OR 1.1, 95% CI 1.0–1.3, *P* = 0.024), D-dimer level (OR 1.1, 95% CI 1.0–1.2, *P* = 0.034) and MB ≥ 1,000 ng/ml (OR 6.5, 95% CI 2.2–19.1, *P* < 0.001) were all independent risk factors for AKI induced by EHS. CK ≥ 1,000 U/L (OR 0.6, 95% CI 0.2–1.9, *P* = 0.421) was not an independent risk factor for AKI (Table 2).

**Table 2:** Risk factors for EHS complicated with AKI.

	**Univariate**	**Multivariate**
	**OR (95%CI) *P*-value**	**OR (95%CI) *P*-value**
Lymphocyte	1.2 (1.0, 1.6) 0.075	2.2 (1.2, 3.4) 0.005
Neutrophil	1.1 (1.0, 1.1) 0.040	1.1 (1.0, 1.3) 0.024
PT	1.1 (1.0, 1.1) 0.004	1.0 (1.0, 1.0) 0.274
FIB	0.8 (0.6, 1.1) 0.239	1.3 (0.9, 1.9) 0.172
D-Dimer	1.1 (1.1, 1.2) <0.001	1.1 (1.0, 1.2) 0.034
**CK** **≥** **1,000 U/L**		
No	1.0	1.0
Yes	1.5 (0.8, 2.7) 0.219	0.6 (0.2, 1.9) 0.421
**MB** **≥** **1,000 ng/ml**		
No	1.0	1.0
Yes	6.8 (3.3, 14.3) <0.001	6.5 (2.2, 19.1) <0.001
**DIC**		
No	1.0	1.0
Yes	3.9 (1.9, 8.2) <0.001	3.5 (0.9, 14.0) 0.076

### Comparisons of Survivors and Non-survivors With EHS Complicated With AKI

Among the patients with AKI induced by EHS, 60 survived (73.2%) and 22 died (26.8%). Non-survivors had higher APACHE II and SOFA scores at admission [22.0 (19.0–23.5) vs. 12.0 (8.0–16.0), *P* < 0.001; 12.0 (9.5–14.5) vs. 4.0 (2.0–6.0), *P* < 0.001] and lower GCS scores [5.0 (3.0–7.0) vs. 12.0 (8.0–14.0), *P* < 0.001]. In the non-survivor group, organ dysfunction was more severe than that in the survivor group; for example, the platelet count was significantly decreased (*P* < 0.001), but the total bilirubin, troponin I, ALT, AST, CK and CK-MB were significantly increased (all *P* < 0.05). Furthermore, the non-survivor group had worse blood coagulation function (PT, INR, D-dimer, all *P* < 0.001) and the higher rates of DIC (94.7 vs. 33.3%, *P* < 0.001) and acute liver injury (100.0 vs. 74.6%, *P* = 0.012) than the survivor group. There was no significant difference in the incidence of lymphocytopenia (*P* = 0.068) and the length of ICU time (*P* = 0.794) between survivors and non-survivors. In addition, the total hospitalization costs in the non-survivor group was significantly higher than that in the survivor group [185,179.7 vs. 46,394.0 (RMB), *P* = 0.003] (Supplementary Table 1).

### Risk Factors for 90-Day Mortality in EHS Complicated With AKI Patients

**Figure 2:**
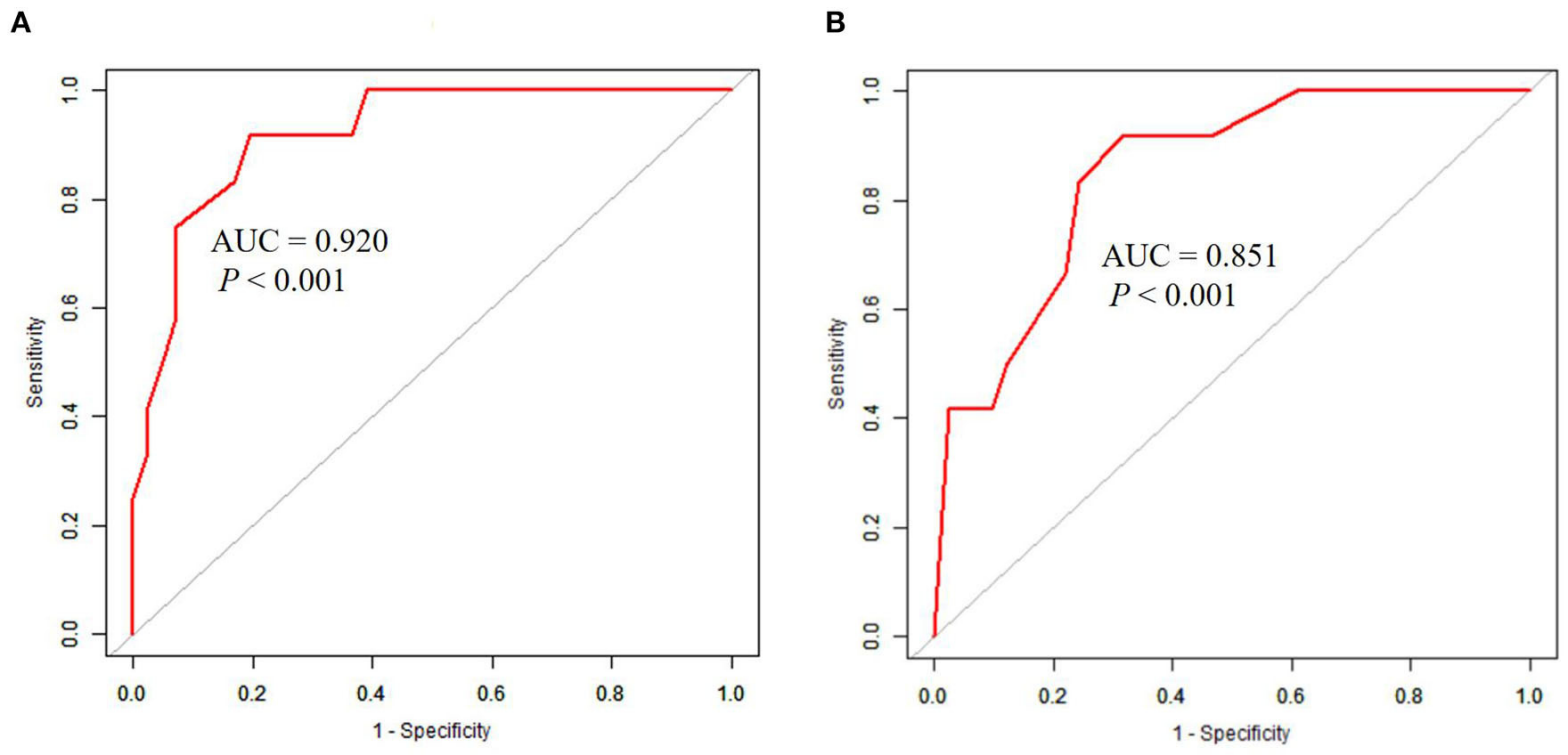
ROC curves in predicting 90-day mortality with AKI patients induced by EHS. **(A)** The AUC of SOFA score was 0.920 (95%CI 0.842–0.998, *P* < 0.001), the optimal cutoff was 7.5 scores, the sensitivity was 91.7%, and the specificity was 80.5%. **(B)** The AUC of GCS score was 0.851 (95%CI 0.739–0.962, *P* < 0.001), the optimal cutoff was 8.5 scores, the sensitivity was 91.7%, and the specificity was 68.3%.

The univariate analysis showed that the APACHE II score, the SOFA score, the GCS score, MB ≥ 1,000 ng/ml, the presence of DIC, the cystatin C level, INR, FIB level, and the D-dimer level were closely related to 90-day mortality in patients with AKI (all *P* < 0.001). Multivariate logistic regression showed that the SOFA score (HR 4.1, 95% CI 1.6–10.8, *P* = 0.004), GCS score (HR 3.2, 95% CI 1.2, 8.4, *P* = 0.017) were an independent risk factor for 90-day mortality in patients with EHS complicated with AKI (Table 3). The AUCs for the prediction of mortality based on the SOFA score (Figure 2A) and the GCS score (Figure 2B) were 0.920 (95% CI 0.842–0.998, *P* < 0.001) and 0.851 (95% CI 0.739–0.962, *P* < 0.001). The optimal cutoff were 7.5 points and 8.5 points, with the sensitivity and specificity were 91.7, 91.7% and 80.5, 68.3%, respectively. Besides, the SOFA score and the GCS score were both significant differences in EHS patients with different AKI stages (*P* < 0.001) (Supplementary Table 2).

**Table 3:** Risk factors for 90-day mortality in EHS complicated with AKI patients.

	Univariate^*^	**Multivariate**
	**HR (95%CI) *P*-value**	**HR (95%CI) *P*-value**
APACHE II score	1.3 (1.2, 1.4) <0.001	1.5 (0.9, 2.5) 0.102
SOFA score	1.5 (1.3, 1.7) <0.001	4.1 (1.6, 10.8) 0.004
GCS score	0.7 (0.5, 0.8) <0.001	3.2 (1.2, 8.4) 0.017
MB ≥ 1,000 ng/ml	7.7 (3.0, 19.9) <0.001	7.5 (0.3, 201.2) 0.230
CK ≥ 1,000 U/L	2.1 (0.9, 4.9) 0.106	0.0 (0.0, 0.4) 0.031
DIC	21.3 (4.9, 92.0) <0.001	83.9 (0.2, 36,555.1) 0.153
Cystatin C	1.8 (1.4, 2.4) <0.001	1.5 (0.6, 3.9) 0.399
INR	1.3 (1.2, 1.5) <0.001	0.3 (0.1, 0.7) 0.007
FIB	0.3 (0.1, 0.4) <0.001	0.0 (0.0, 0.1) 0.003
D-dimer	1.0 (1.0, 1.1) <0.001	1.0 (0.9, 1.0) 0.175
Acute liver injury	9.6 (1.3, 71.4) 0.027	inf. (0.0, Inf) 0.998

* *Adjust model adjust for: Age*.

**Figure 3:**
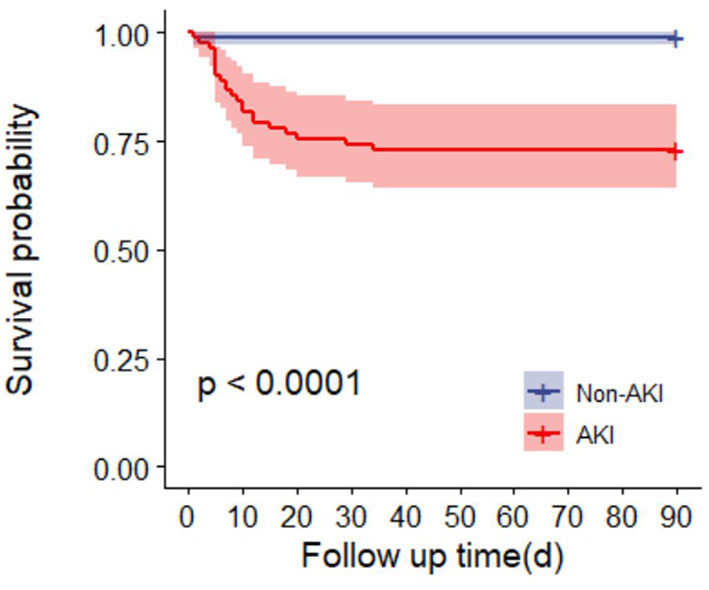
Survival curves of 90-day mortality rate in AKI group and non-AKI group.

**Figure 4:**
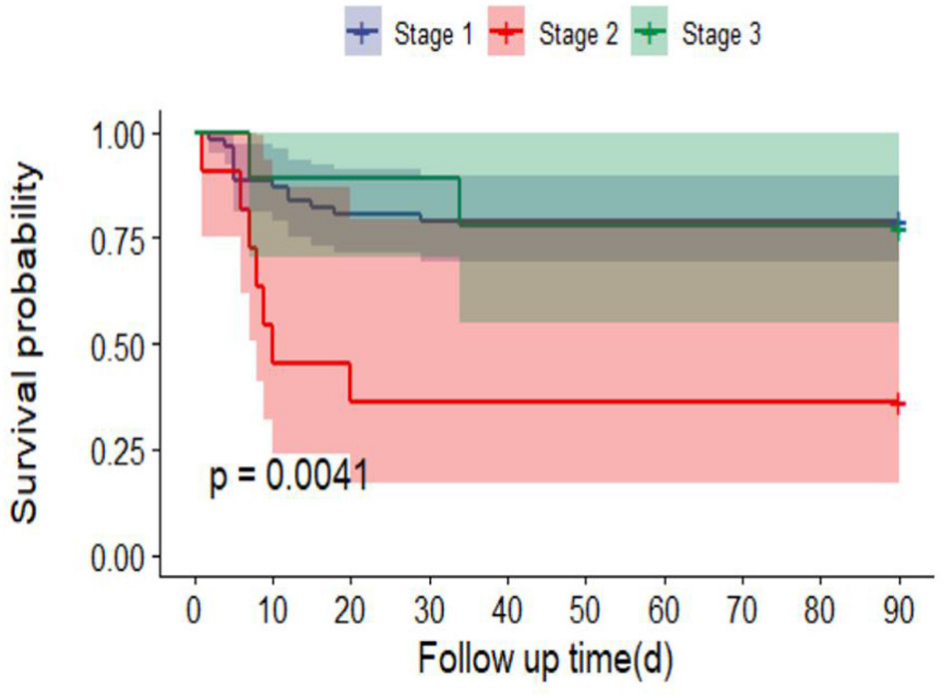
Survival curves of 90-day mortality rate in different AKI stages.

Ninety-day mortality in AKI patients was significantly increased after adjusting for RM (CK ≥ 1,000 U/L or MB ≥ 1,000 ng/ml) (*P* < 0.0001) (Figure 3). In addition, 90-day mortality was different in patients with different stages of AKI; mortality in patients with stage 2 AKI was the highest (*P* = 0.0041) (Figure 4).

## Discussion

### The Main Findings of This Study

This retrospective cohort study included the largest number of clinical cases of EHS complicated with AKI in southern China. In this study, we observed that 43.9% of patients with EHS developed AKI, and 26.8% of AKI patients died within 90 days. Multivariate logistic regression showed that the lymphocyte count, neutrophil count, D-dimer level and MB ≥ 1,000 ng/ml were all independent risk factors for AKI induced by EHS. Moreover, both of the SOFA score and GCS score at admission were independent risk factors for 90-day mortality in patients with AKI induced by EHS. Compared with non-AKI patients, AKI patients developed more severe organ injury, and had a higher incidence of MB ≥ 1,000 ng/ml, DIC and acute liver injury. There was a significant increase in 90-day mortality and hospitalization costs in this group. Furthermore, it was found that the survival time was different with different AKI stages; interestingly, 90-day mortality in patients with AKI stage 2 was the highest.

### The Underlying Pathogenic Mechanisms of AKI Induced by EHS

The underlying pathogenic mechanisms include reduced blood volume (10), microthrombi in renal afferent and efferent arteries (11), blockage of the renal tubules by MB or direct nephrotoxicity, and renal interstitial cell inflammation (12). AKI is closely related to macrophages (13, 14) as well as dendritic cells (15) in the renal interstitium. As all the patients in this study were young men who were in good health and did not have basic diseases such as kidney disease, heart disease or lung diseases, organ injury was considered to be caused by EHS.

### The Importance of Inflammation and Coagulation Dysfunction

The multivariate analysis found that inflammation (lymphocytes, neutrophils) and coagulation dysfunction (D-dimer) were closely related to EHS with AKI. A previous retrospective study (16) evaluated 176 patients with heatstroke (aged from 19 to 76 years, with an average age of 47.5 years in the AKI group and 39.6 years in the non-AKI group). The results showed that the lowest platelet count (OR 37.92, 95% CI 2.18–87.21, *P* < 0.01) was an independent predictor of AKI in patients with heatstroke. This was not consistent with the results of our research. Some reasons may be related to the characteristics of the study populations, the cause of overheating or the environmental humidity in the study location. The average median age of our study population was 21 years old. All of the patients experienced overexertion, had no underlying diseases and had good physiological reserve. In these patients, the host immune response was strong, and the activation of inflammatory cells and endothelial injury led to the development of AKI (17–19). Moreover, AKI can happen from EHS-induced tissue rupture, myoglobinuria, hypovolemia and metabolic acidosis (20). Thus, in this population with heatstroke, reducing inflammatory reactions may reduce organ injury. Our previous studies showed that ulinastatin can protect against severe bowel, lung, heart and other organ damage (21, 22). Recent studies have shown that ulinastatin alleviates AKI caused by inflammation in crush syndrome by regulating Th17/Treg cells (23). As a result, treatments targeting inflammation and blood coagulation may be clinically significant in studies on kidney injury caused by EHS in the future.

### MB: A Better Biomarker of RM

The multivariate results also showed that MB ≥ 1,000 ng/ml was an independent risk factor for AKI caused by EHS, while CK ≥ 1,000 U/L was not. The criterion for diagnosing RM in EHS using CK > 1,000 U/L is, therefore, too broad. Some studies (24) have reported that CK > 10,000 U/L (50 times the normal value) is valuable in predicting renal failure in patients with EHS. In Gearoid M's study (25), which included 2,371 patients, CK > 5,000 U/L was considered the diagnostic criterion. It was found that the results of RM varied with clinical etiology, and the prognosis of RM could be predicted by common demographic characteristics and clinical and laboratory variables. The authors believe that the CK cutoff value is excessively strict, and it is easy to omit the diagnosis in patients with stage 1–2 kidney injury. What's more, CK increases in the first 12 h, peaks on the second or third day, and returns to baseline after 3–5 days. While the half-life of MB is 2–3 h, and serum MB levels may return to normal within 6–8 h (26). It is of theoretical and practical significance to establish MB as a biomarker of RM and its cutoff value in patients with EHS. In addition, the univariate analysis showed that the APACHE II score, SOFA score, GCS score, cystatin C level, MB ≥ 1,000 ng/ml, INR, FIB level and D-dimer level were all associated with the 90-day prognosis in these patients, and all of them were statistically significant. The OR (3.3) for MB ≥ 1,000 ng/ml was the highest. Hence, our study indicated that the main cause of death may be elevated MB (≥1,000 ng/ml), suggesting that a high concentration of MB in serum has the potential to influence renal damage. Because elevated MB usually signifies a higher degree of muscle damage that may lead to liver and kidney complications that could result in the death of the patient or AKI (26). As a result, an in-depth study of the pathological mechanism of MB elevation in the kidneys and a clinical treatment strategy based on reducing MB may provide a new theoretical basis and potential treatment strategies for kidney injury caused by EHS with RM.

### SOFA Score: An Independent Risk Factor for 90-Day Mortality

Multivariate logistic regression showed that only the SOFA score was an independent risk factor for 90-day mortality in EHS patients with AKI. Cheng's study (27) compared the SOFA score, APACHE II score and simplified acute physiology score (SAPS) II and found that the SOFA score was most accurate in predicting 28-day mortality in patients with heatstroke, with a cutoff of 9.0, a sensitivity of 84.6%, and a specificity of 71.1%. This is primarily consistent with our previous studies and Kondo's studies (28). Early treatment strategies based on altered SOFA scores may be an effective measure to reduce 90-day mortality. Previous studies have suggested that with the progression of AKI, the mortality rate increases gradually. However, our data showed that patients with stage 2 AKI (KDIGO criteria) had significantly shorter survival than patients with other stages. The underlying mechanism may be that EHS leads to severe injury in other organs, such as the brain, or DIC, which has a greater impact on 90-day mortality than AKI. These results are different from those associated with sepsis or surgery (29–31). But this result still needs a large sample study to be confirmed in the future.

In this study, renal function returned to normal at discharge in all patients except non-survivors, which meant that they did not develop into chronic kidney disease (CKD). However, a retrospective study included 2,529 EHS patient of US military showed that some patients had persistent kidney damage or dysfunction (32), which was different from our study. The underline influence factors may be related to age. Other factors also including the treatment strategy of first aid and the time from onset to first aid site. Due to our patients developed EHS in different places at different times, and the distance between the site of EHS happened and the first aid site was varied, many patients in our study didn't receive immediately ice water cooling treatment, while DeMartini et al. (33) shows that when immediate ice water cooling could save the patients from death induced by EHS, which is one of the key cornerstones to reduce the short-term and long-term adverse outcomes of heatstroke. Therefore, using whole-body cold water immersion immediately is the proper treatment protocols of EHS in first aid site, and only in this way, the patients are not likely to develop AKI and problems associated with CKD.

### Limitations of This Study

This study has limitations. This was a single-center retrospective cohort study with a relatively small number of cases. In addition, all the patients were male, the average age was relatively young, and the type of heatstroke was restricted to EHS. The results do not fully reflect the overall conditions of the heatstroke population, which may reduce the reliability of the statistical analysis. Subsequent studies should expand the sample size and employ a prospective randomized controlled trial design to achieve higher-level clinical results.

## Conclusions

EHS patients with AKI had a worse clinical condition, a significantly higher total cost of hospitalization and significantly higher 90-day mortality than those without AKI. MB ≥ 1,000 ng/ml was an important independent risk factor for AKI in patients with EHS. Early treatment strategies based on altered SOFA scores may be an effective measure to reduce 90-day mortality in these patients.

## Data Availability Statement

The datasets presented in this article are not readily available because should sent an e-mail to corresponding author to explain the purpose. Requests to access the datasets should be directed to Zhifeng Liu, zhifengliu7797@163.com.

## Ethics Statement

The studies involving human participants were reviewed and approved by the Research Ethics Committee of the General Hospital of Southern Theatre Command of PLA (HE-2020-09). Written informed consent for participation was not required for this study in accordance with the national legislation and the institutional requirements.

## Author Contributions

ZhiL, BC, and MW were responsible for study concept and design. MW, CW, ZheL, LZ, and BY were responsible for collecting the data. MW and CW were responsible for statistical analysis. ZhiL, MW, and CW were responsible for drafting the manuscript. All authors had full access to all the data in the study and take responsibility for the integrity of the data and the accuracy of the data analysis.

## Conflict of Interest

The authors declare that the research was conducted in the absence of any commercial or financial relationships that could be construed as a potential conflict of interest.
