# Supplementary material for: Clinical Characteristics and Risk Factors Associated With Acute Kidney Injury Inpatient With Exertional Heatstroke: An Over 10-Year Intensive Care Survey
Source: Front Med (Lausanne). 2021 May 19;8:678434. doi: 10.3389/fmed.2021.678434 (PMC8170299; doi:10.3389/fmed.2021.678434)
Supplement: Supplementary file 1 [file Table_1.pdf]

SUPPLEMENT TABLE 1 Comparisons of clinical characteristics between survivors and non-survivors with EHS complicated with AKI

	**Survivor (n = 60)**	**Non-survivor (n = 22)**	***P*-value**
APACHE II score, median (IQR)	12.0 (8.0-16.0)	22.0 (19.0-23.5)	<0.001
SOFA score, median (IQR)	4.0 (2.0-6.0)	12.0 (9.5-14.5)	<0.001
GCS score, median (IQR)	12.0 (8.0-14.0)	5.0 (3.0-7.0)	<0.001
Age (years), median (IQR)	23.5 (19.8-31.0)	21.0 (18.0-24.0)	0.084
WBC (1×10^9/L), median (IQR)	12.0 (9.8-15.6)	10.6 (8.4-15.7)	0.614
Neutrophil (1×10^9/L), median (IQR)	9.6 (6.8-13.3)	8.9 (6.6-13.2)	0.814
Lymphocyte (1×10^9/L), median (IQR)	1.1 (0.6-2.3)	0.6 (0.3-2.6)	0.554
Monocytes (1×10^9/L), median (IQR)	0.7 (0.4-1.0)	0.7 (0.3-0.9)	0.705
Platelets (1×10^9/L), median (IQR)	163.0 (79.5-215.5)	72.0 (29.0-89.0)	<0.001
Mean platelet volume (%), median (IQR)	10.7 (10.2-11.3)	11.0 (10.4-11.8)	0.141
Platelet distribution width (%), median (IQR)	12.3 (11.6-13.5)	13.2 (12.2-15.8)	0.023
TBIL (μmol/L), median (IQR)	14.6 (9.5-27.7)	29.9 (12.9-122.9)	<0.001
ALT (U/L), median (IQR)	36.0 (22.0-215.0)	220.0 (87.0-1771.0)	0.025
AST (U/L), median (IQR)	73.0 (39.0-197.5)	361.5 (106.5-1757.5)	0.017
BUN (mmol/L), median (IQR)	6.8 (5.6-10.1)	8.1 (6.2-9.5)	0.858
CR (μmol/L), median (IQR)	160.0 (146.8-192.8)	222.0 (184.0-274.0)	0.510
Cystatin C (mg/L), median (IQR)	1.2 (1.0-1.6)	1.5 (1.1-3.0)	0.116
CK (U/L), median (IQR)	884.0 (441.5-2954.2)	1,672.0 (848.5-7324.5)	0.042
CK-MB (ng/ml), median (IQR)	39.0 (29.0-72.0)	102.0 (47.0-348.0)	0.025
MB (ng/ml), median (IQR)	973.0 (405.1-1000.0)	1,000.0 (861.6-1000.0)	0.315
cTNI (pg/ml), median (IQR)	190.0 (60.0-486.0)	1,320.0 (871.9-2885.0)	<0.001
PT (s), median (IQR)	16.1 (14.1-22.2)	35.0 (23.5-44.4)	<0.001
INR, median (IQR)	1.3 (1.1-1.9)	3.6 (2.1-4.9)	<0.001
APTT (s), median (IQR)	36.6 (31.6-49.6)	86.1 (65.9-120.9)	<0.001
TT (s), median (IQR)	17.6 (16.6-20.9)	36.4 (26.6-58.8)	0.041
FIB (g/L), median (IQR)	2.4 (2.1-2.9)	1.4 (0.9-2.0)	0.002
D-D (mg/L), median (IQR)	3.0 (0.9-7.6)	10.1 (10.0-20.0)	<0.001
CRP (mg/dL), median (IQR)	3.3 (0.6-9.6)	3.4 (3.3-3.6)	0.484
PCT (ng/ml), median (IQR)	2.6 (1.1-5.1)	3.3 (1.5-7.1)	0.322
CK ≥ 1000 U/L, N (%)	27/56 (48.2%)	13/20 (65.0%)	0.179
MB ≥ 1000 ng/ml, N (%)	24/49 (49.0%)	14/20 (70.0%)	0.111
Transfusion, N (%)	20/59 (33.9%)	15/16 (93.8%)	<0.001
Lymphocytopenia < 0.8×10^9/L, N (%)	22/60 (36.7%)	13/21 (61.9%)	0.068
DIC, N (%)	15/45 (33.3%)	18/19 (94.7%)	<0.001
Acute Liver Injury, N (%)	44/59 (74.6%)	21/21(100.0%)	0.012
ICU time (d), median (IQR)	7.0 (4.0-14.0)	8.0 (5.0-12.0)	0.794
Hospitalization costs (RMB), median (IQR)	46,394.0 (36,747.1-126,369.3)	185,179.7 (152,352.0-379,098.0)	0.003
