# Supplementary material for: Clinical Characteristics and Risk Factors Associated With Acute Kidney Injury Inpatient With Exertional Heatstroke: An Over 10-Year Intensive Care Survey
Source: Front Med (Lausanne). 2021 May 19;8:678434. doi: 10.3389/fmed.2021.678434 (PMC8170299; doi:10.3389/fmed.2021.678434)
Supplement: Supplementary file 2 [file Table_2.pdf]

SUPPLEMENT TABLE 2 Comparisons of SOFA and GCS score in EHS patients with different AKI stages

	**Non-AKI (n = 105)**	**AKI Stage 1 (n = 62)**	**AKI Stage 2 (n = 11)**	**AKI Stage 3 (n = 9)**	***P*-value**
SOFA score	2.0 (2.0-4.0)	4.0 (2.2-6.0)	14.0 (9.5-14.5)	11.0 (10.0-11.5)	<0.001
GCS score	12.0 (9.0-14.0)	11.0 (7.0-14.0)	5.0 (3.5-6.0)	10.5 (7.5-12.5)	<0.001
